# Designing a Local Policy to Reduce HIV in Mexico City

**DOI:** 10.1055/s-0043-1773791

**Published:** 2023-08-29

**Authors:** Mayar Al Mohajer

**Affiliations:** 1 Department of Medicine, Section of Infectious Diseases, Baylor College of Medicine, Houston, Texas, United States

**Keywords:** HIV, Mexico, strategy, prevention, early treatment

## Abstract

The Joint United Nations Program on human immunodeficiency virus/acquired immunodeficiency syndrome (HIV/AIDS) (Joint United Nations Program on HIV/AIDS, UNAIDS) has recommended 90–90–90 goals to increase the number of patients who are aware of their status, on antiretroviral therapy, and have undetectable viral loads. Mexico City has made several achievements to aid in prevention, early diagnosis, and treatment; however, the incidence of HIV has not decreased over the past decade. This article reviews global initiatives that were successful in achieving some or all these metrics and provide a road map for Mexico to reach the desired goals.

## Introduction

Around 340,000 individuals are currently living with human immunodeficiency virus (HIV) in Mexico including over 40,000 in Mexico City. 1 2 According to the Joint United Nations Program on HIV/AIDS (UNAIDS), 55% of people with HIV in Mexico are receiving antiretroviral therapy (ART) and 49% have undetected viral load. 1 In 2019, there was an estimated 1,313 new HIV diagnoses in Mexico City (16,893 nationwide and 1,989,000 globally), with an incidence of 14.9 cases per 100,000 (13.5 nationwide and 25.7 globally). 3 Around 420 deaths were caused by HIV in Mexico City in 2019. 3 It is important to note that the numbers present here for incidence and prevalence are estimates generated by UNAIDS 3 and are significantly higher than the official numbers 2 indicating differences in data collection and missed cases. Relying on official numbers alone could lead to underestimating the proper efforts needed to reach the targeted goals.

Younger men in Mexico City are more affected by HIV than the general population. For example, in 2019 men aged between 25 and 39 years were four times more likely to live with HIV (prevalence 0.74–0.81%) compared with the average population (prevalence 0.21). 3 Moreover, the incidence is higher in people aged between 15 and 39 years, peaking at 85 cases per 100,000 population in people aged between 20 and 24 years, versus 5.7 for the national average. 3

Several key groups are disproportionately impacted by HIV in Mexico. The population of men who have sex with men (MSM) is estimated to be 1,200,000 with an HIV prevalence of 11.9% (63 times higher than national average of 0.19%). 1 This figure represents 42% of all HIV cases in the country. 1 Other key groups include transgender people, people who inject drugs, sex workers, and prisoners. 1

**Fig. 1:**
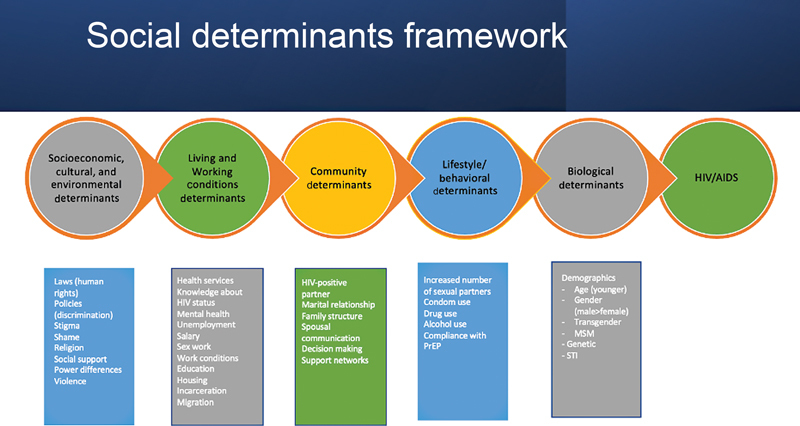
Social determinants network.

Studies in Mexico City have identified the number of sexual partners, inconsistent condom use, HIV-positive partner, and injection drug use as major risk factors for HIV. 4 5 6 Several socioeconomic factors were demonstrated to increase HIV acquisition there including lower education, 4 5 6 lower salary, 5 migration, 5 spousal communication, 5 and mental health. 7 Fig. 1 shows a more comprehensive framework of social determinants based on literature review.

Mexico City has made great strides in its current response to fight the HIV pandemic leading to lower HIV incidence and prevalence than the global figures. The proportion of HIV-positive patients on ART has increased from 28% in 2010 to 55% in 2020. Death rate has been steadily dropping in Mexico City from a peak of 11.5 deaths per 100,000 in 1996 to 4.8 in 2019. 8 This progress has followed several initiatives in diagnosis (propagating HIV testing), treatment (centralized purchase of ART), and viral suppression (viral load test purchasing and monitoring). 8 However, more efforts are needed to further reduce the health impact of HIV/AIDS in Mexico City.

## Policy Importance

The UNAIDS has published 2020 global goals 9 for HIV testing and treatment including (1) 90% of HIV-positive individuals aware of their status, 90% of them on ART, and 90% of them virally suppressed (90–90–90). In addition, essential prevention efforts, such as condom use and pre-exposure prophylaxis (PrEP), should be scaled up. Reaching these goals requires partnering of financing, advocacy/ policy, normative/ technical, and research and development sectors. 9 If implemented, these goals will be able to end the pandemic by 2030.

Despite the aforementioned response efforts in Mexico City, the UNAIDS goals have not been met 1 and the HIV incidence has gradually increased over the past two decades. Hence, a local policy is urgently needed. While the mortality rate has decreased from 5.25 deaths per 100,000 in 2014 to 4.76 in 2019, the estimated number of HIV/AIDS-related deaths and the rate of years lost to disability from HIV/AIDS have remained stable. 3

## Methods

Country profiles from UNAIDS were utilized to determine which countries met their 90/90/90 goals. Countries that met at least one target were included in the search. Medline database was used to search for global initiatives in the selected countries to control HIV. Key search terms included HIV, AIDS, 90–90–90, testing, prevention, early treatment and undetected, UNAIDS, goals, and Miles to Go global strategy. Policy options were developed based on the local context of Mexico City. Metrics were established to ensure policy options are implemented and monitored over a 5-year period.

## Results and Discussion

### Policy Suggestions

According to the UNAIDS Miles to Go global strategy, 10 local priorities in locations with low-medium incidence of HIV should focus on both the left-behind at-risk populations (MSM, transgender people, sex workers and their clients, HIV-positive patients, intravenous drug users, and their partners) and lower risk ones (population of reproductive age, young people, and adolescents). Henceforth, our local plan should have a hybrid approach to attain the global goals in Mexico City.

Various approaches were demonstrated effective in reaching the UNAIDS goals in countries with similar economic challenges as the ones Mexico experiences. Shifting from hospital-based PrEP programs to mobile clinics was advantageous in expanding coverage in South Africa. 11 Home-based HIV testing and counseling with a couple-focus approach were used in countries in Southern Africa and India. 12 In Eswatini, a remarkable level of linkage to care (97%) was achieved when community testing was combined with case management, psychological assistance, accompanying patients to appointments, financial support for transportation, and appointment reminder and navigation. 13 Providing antiretroviral directly observed therapy (DOT) was successful in achieving undetectable viral load goal in Boston, 14 which is similar in that both Boston and Mexico City are large urban centers.

### Key Targets and Initiatives

Based on the previous review of literature, several targets and initiative are suggested for the government to follow over the next 5 years including the following:

1. Fund and establish adequate mobile clinics to reach at-risk groups and provide PrEP in all 16 districts of Mexico City. It is recommended that clinics use the bimonthly injectable form that has recently been approved by the Food and Drug Administration in the United States (unless the patient refuses), rather than the oral option, given higher rates of compliance and efficacy since the first is given bimonthly versus daily for the second. 15 Districts should have a target of 25% of MSM and transgender people, 18% of sex workers and people who inject drugs, and 0.4% of heterosexual adults aged between 15 and 59 years receiving PrEP in the next 5 years. These targets were derived from the Centers for Disease Control and Prevention (CDC) estimates for PrEP need per at-risk group. 16
2. Expand HIV testing program by deploying community workers to perform a one-time home-based point-of-care rapid testing to cover 90% of Mexico City residents aged between 15 and 59 years and increase the rate of people living with HIV who are aware of their status to 90%. Testing should be done utilizing participatory approaches with community partners to ensure reaching out to all the left behind groups.
3. Develop community-based linkage programs providing financial, psychological, social, and logistical support to newly diagnosed HIV patients and provide same-day ART to them. Districts should have a target of 90% of all HIV cases diagnosed through community testing be seen for appointment and started on ART within 90 days from diagnosis.
4. Launch a DOT program that provides ART regimens for all HIV-positive patients. Injectable monthly regimens will be preferred to improve compliance 17 ; however, daily oral options will remain available if a patient declines. Districts should provide DOT for 90% of patients on ART and should have 90% of all ART-receiving patients achieving undetectable viral load within 3 months of initiation.

## Key Strengths, Risks, and Limitations

*Strengths* : The policy suggestions identified here are consistent with the UNAIDS global plan and target the at-risk populations. They build on successful interventions in similar locations (low- and middle-income countries or large urban areas) that were able to reach the 90–90–90 goal. There are some similarities between those plans and the one presented here in terms of key populations targeted (as in Boston) and financial challenges (as in India and Southern Africa). Also, by using injectable ART and PrEP (approved by the United States Food and Drug Administration on January 21, 2021, 18 and December 20, 2021, 15 respectively), the number of users acquiring HIV will reduce given the convenience less frequent administration entails and the potency of their components.

*Risks* : There are concerns about the considerable financial and labor resources needed to accomplish the goals. However, modeling data provided by UNAIDS 10 have shown the cost effectiveness of similar programs. Another concern is the potential increase in disparity if prevention and treatment efforts fail to include disadvantaged groups. Participatory approaches should be done to engage these communities during the designing, performing, and monitoring phases of this policy to ensure success. Difficulty reaching out to the affected residents due to coronavirus disease 2019 and general safety concerns in some Mexico City neighborhoods 19 and pre-existing ART shortage 20 are additional challenges that could further prevent escalating screening and treatment efforts.

*Limitations* : The interventions proposed here were based on experiences from other countries. While risk factors for HIV acquisition are similar across the globe, determinants of health differ among countries limiting data generalization. For example, heterosexual transmission is more common in Africa than in Mexico City. Boston has significantly more financial resources and access to newer technology than Mexico City. The estimates for PrEP coverage were based on CDC risk assessment of at-risk groups in the United States and might not be applicable to individuals in Mexico City due to possible differences in risk engagement. In addition, our proposed interventions are not exhaustive of all possible options and solutions.

## Measurement and Evaluation

To measure and evaluate the effectiveness of the local strategic plan, districts should collect quarterly data and outcomes for each of the proposed interventions ( Table 1 ). In addition, data will be collected to assess the impact of these efforts on the estimated incidence, prevalence of HIV and AIDS, and its associated mortality. The results will be stratified by district, age-group, gender, and at-risk category.

**Table 1:** Measurement and evaluations for key interventions

Interventions	How to collect data	Indicators	Goals
Mobile clinics to provide PrEP	- Online and face-to-face surveys to accurately predict the demand based on the estimated number of at-risk individuals - Obtain data from mobile clinics on time from identification to PrEP dispensation and number of prescriptions written	- Wait time between individual identification and PrEP dispensation (process)	- Wait time less than 30 days
		- Percentage of at-risk individuals covered by PrEP per at-risk group (outcome)	- Percentage covered ≥ 25% MSM and transgender people, 18% sex workers and IVDU, and 0.4% heterosexuals aged 15–59
Expand HIV testing	- Online and face-to-face surveys to estimate the number of individuals 15–59 with an HIV test performed over their lifetime - Generate estimates of people living with HIV who are aware of their status (currently not measured in Mexico)	- Percentage of individuals aged 15–59 with an HIV test ever performed (process)	- Percentage tested ≥ 90%
		- Percentage of people living with HIV who are aware of their status (outcome)	- Percentage aware ≥ 90%
Develop community-based linkage programs	- Obtain data from community testing centers on the number of new HIV cases diagnosed - Obtain data from HIV clinics on the number of new appointments, time to visit, the number of new HIV cases started on ART, and time to ART initiation	- Time to visit and time to ART initiation (process)	- Wait time < 90 days from diagnosis
		- Percentage of newly diagnosed patients started on ART within 90 days (outcome)	- Percentage started on ART within 90 days ≥ 90%
Launch a DOT program	- Obtain statistics from HIV clinics on the number of patients on ART receiving DOT, and their laboratory results (CD4 and viral load)	- Percentage of patients on ART receiving DOT (process)	- Percentage receiving DOT ≥ 90%
		- Percentage of patients on ART becoming undetectable within 3 months of diagnosis (outcome)	- Percentage undetectable ≥ 90%

Abbreviations: ART, antiretroviral therapy; DOT, directly observed therapy; HIV, human immunodeficiency virus; IVDU, intravenous drug users; MSM, men who have sex with men; PrEP, pre-exposure prophylaxis.

## Conclusion

We created several policy options for Mexico City that could aid in reaching the UNAIDS goals of 90–90–90. They included expanded HIV testing and PrEP through mobile clinics and community workers, a community linkage, and a DOT program for ART. New injectables are key to increase compliance with PrEP and ART. Measurement and evaluation indicators should be used to ensure success.
